# Yellowstone Wolves and the Forces That Structure Natural Systems

**DOI:** 10.1371/journal.pbio.1002025

**Published:** 2014-12-23

**Authors:** Andy P. Dobson

**Affiliations:** 1 Department of Ecology and Evolutionary Biology, Princeton University, Princeton, New Jersey, United States of America; 2 Santa Fe Institute, Santa Fe, New Mexico, United States of America

## Abstract

Since their introduction in 1995 and 1996, wolves have had effects on Yellowstone that ripple across the entire structure of the food web that defines biodiversity in the Northern Rockies ecosystem. Ecological interpretations of the wolves have generated a significant amount of debate about the relative strength of top-down versus bottom-up forces in determining herbivore and vegetation abundance in Yellowstone. Debates such as this are central to the resolution of broader debates about the role of natural enemies and climate as forces that structure food webs and modify ecosystem function. Ecologists need to significantly raise the profile of these discussions; understanding the forces that structure food webs and determine species abundance and the supply of ecosystem services is one of the central scientific questions for this century; its complexity will require new minds, new mathematics, and significant, consistent funding.

## Abstract 2

The reintroduction of wolves to Yellowstone has provided fascinating insights into the ways species interactions within food webs structure ecosystems. Recent controversies about whether wolves are responsible for all observed changes in prey and plant abundance suggest that we need many more such studies, as they throw considerable light on the forces that structure the parts of the universe that are of vital importance to humans.

In the half moonlight at dawn on a sharply cold January morning, they looked like small ponies galloping beside the old railroad at the northern entrance of Yellowstone National Park. They weren't ponies. This was the “Eight Mile” wolf pack, each member huge, healthy, and vigorous, romping through the light snow on a morning quest for elk, bison, or anyone too slow to get out of their way. It was an incredible moment, one that evoked feelings shared by the hundreds of wolf watchers who come to Yellowstone every month of the year hoping to experience even a glimpse of the wolves.

The enthusiasm of the wolf watchers is almost totally reversed by the many local ranchers who live outside the park and regard wolves as varmints, best used for target practice. The pack that I heard howling outside the cabin every night was quickly dispatched by the local rancher soon after I left; their pelts could be found for sale in one of the souvenir stores at the entrance to Yellowstone.

Scientists initially appear as polarized in their opinions of the role of wolves and large predators in ecosystems as the wolf watchers and ranchers are about their value to the local economy. Wolves were introduced back into Yellowstone following the development of a huge environmental impact assessment (EIA) that attempted to predict the outcome of their reintroduction. The EIA, a 4-ft-deep pile of documents, provided solid testimony to the need for a deeper empirical and theoretical understanding of how ecological food webs respond to species additions and losses. At the time, even the suggestion of introducing wolves created huge discord in the ranching community surrounding Yellowstone; most ranchers (and some ecologists) were convinced wolves would feed exclusively on cattle and sheep; the ranching industry was dead set against reintroduction. A curious event then occurred: photographers started getting photographs of wolves that had naturally colonized the park. As any natural colonization would provide the wolves with full legal protection under the United States Endangered Species Act, the Ranchers Association hastily made a U-turn and supported introduction on the grounds that experimentally introduced wolves were nonnative and could be shot if they left the park. I know of no better environmental example of nonlinear political expediency.

Once introduced in 1995 and 1996, the wolf population grew rapidly. At the time, the elk population was declining from an all-time high and provided a large supply of prey to fuel wolf reproduction; the population increased at close to the maximum rate ever recorded [1]. As the wolf numbers increased, the elk numbers decreased, but at a rate that was more parsimoniously explained by a prolonged drought and levels of human harvest, the decline in abundance far exceeding that which could be accounted for purely in terms of elk consumed by wolves [2],[3]. Significant evidence does suggest that the elk had changed their feeding habits in the presence of wolves, avoiding areas where they could readily be ambushed [3]–[8]. This allowed vegetation in riparian areas to recover; photographs taken at a variety of locations showed considerable recovery of aspen in areas where it had become overgrazed in the years when elk were abundant [1],[9]. Although these riparian areas cover only a small area of the ecosystem (<2%), the park was witnessing the first significant growth of aspen for over half a century. More recent data suggest that similar recoveries are being seen in cottonwoods and willows [1]; this in turn has led to an increase in the abundance and diversity of riparian bird species [10]. All of this evidence suggests that wolves have a strong top-down effect on trophic structure of the ecosystem (Fig. 1).

**Figure 1:**
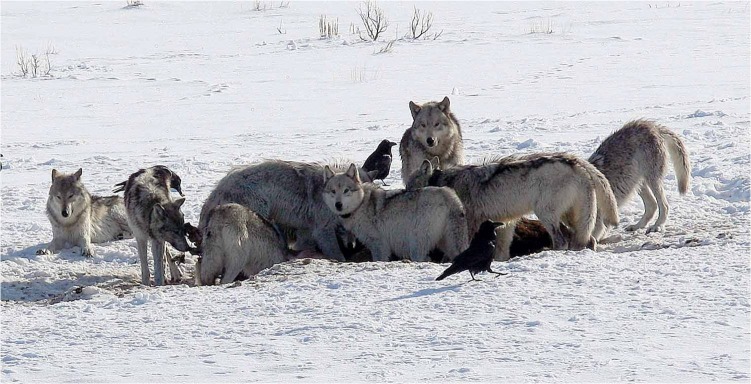
Wolves in Yellowstone NP. Photo credit: Daniel Stahler/National Park Service (NPS) photo from http://www.yellowstonewolf.org/, a site at which information and pictures of Yellowstone wolves can be found.

Alternatively, climate has been argued to be the principal driver of ecosystem change, not wolves; changes in vegetation may have been driven by bottom-up changes in water availability due to changes in snow melt patterns [11]. Wolf population expansion occurred at a time when the Yellowstone region was entering a prolonged drought that also reduced forage available to elk; this combined with human harvest contributed significantly to the declines in ungulate abundance. Furthermore, climate change has lengthened the growing season for willows and aspen by around 27 days in the last couple of decades [12], while the vegetation in many areas of the park is dominated by conifer forest that has simultaneously been recovering from the fires of 1989. Thus, it is not straightforward to differentiate between postfire recovery and the indirect effects of carnivores on vegetation regeneration.

Concomitant to wolf introduction, the grizzly bear population was increasing, creating the potential for indirect competition between bears and wolves as the latter selectively prey on old or injured elk in the winter. This predation reduces the number of elk that would otherwise die and become available for grizzlies emerging from hibernation in the spring. This absence of “frozen meals” caused grizzlies to switch to feeding on elk calves as an alternative spring food source when recovering from their long winter fast [13]. As elk numbers declined following the triple assault of drought, wolves, and bears, both grizzlies and some wolf packs switched their attention to bison [14], which require larger packs to make an effective kill but ultimately provide a larger meal. All of the extra carcasses have provided a new bounty of food for ravens and golden eagles, both of which have increased in abundance [2].

Less well understood is the impact of wolves on coyotes, the numbers of which may have declined since wolves were reintroduced [2]; carnivores are aggressive to other carnivores of similar but slightly smaller body size. As coyotes were the primary predators of sheep, you would think that the sheep ranchers would applaud wolves for the reduced loss of stock to predators; they have been noticeably silent on this front. More subtly, the presence of wolves may help reduce the threat posed by chronic wasting disease (CWD), an emerging prion pathogen that is spreading from elk and deer to cattle and is arguably the biggest biological threat to ranching in the region [15]. Unfortunately, the ranching community does not recognize that the wolves may be doing them a huge favor by removing sick elk and mule deer infected with CWD (and elk and bison infected with brucellosis) from the wild reservoir of infection. If CWD or *Brucella* enters cattle herds in the states bordering Yellowstone, then federal mandates will hugely restrict movement of cattle in and out of these states.

We may have to wait at least another ten years before the impact of wolves on the Yellowstone ecosystem is fully quantified. Although many strong patterns are observed, several of these may be correlation without causation (for example, the increase in beaver abundance is more likely to be a consequence of beaver introductions to the north of Yellowstone National Park [NP] [12]). Furthermore, although there is considerable pressure from the conservation community to sanctify the wolves as bringing only benefits to the ecosystem [12], there is still a need for stronger data to support some of the beneficial claims made for wolf reintroduction. Some of this will come from Yellowstone, but this needs to be combined with studies of wolf reintroduction, or natural reestablishment, in other ecosystems. If the patterns observed in Yellowstone are repeated, as preliminary evidence suggests, then hard-core wolf haters are going to need to reconsider the labelling of wolves as varmints.

The research and debates surrounding the role of wolves in modifying the behavior and abundance of species on multiple trophic levels in Yellowstone illustrates the complex interactions between the forces that structure patterns of abundance in natural ecosystems. The debate gets to the heart of one of the central scientific challenge of ecology: how can we understand the structure of food webs? Central to any discussion of food-web dynamics and ecosystem management is the relative importance of top-down roles played by large predators and pathogens and bottom-up forces driven by the climatological processes that determine plant growth. All of the work from Yellowstone cries out for the development of next-generation, population-based ecosystem models that focus on interactions between climate, vegetation, and the dominant herbivore and carnivore species in the park. In particular, food-web ecologists need to more aggressively move beyond descriptions of the network geometry of food webs and grasp the thistle of food-web dynamics. More generally, we cannot afford for this debate to become polarized; that simply suggests to funding agencies and the general public that ecologists do not know how ecological systems function. Instead, we need to frame the discussion as a major scientific challenge that requires significant international and national funding.

There are curious and unexplored parallels between work on food webs and trophic interactions and that of physicists who are trying to understand the forces that determine the way the universe is structured at either the atomic or astronomical level. At both scales, a series of nested forces hold increasingly large particles together using a mixture of centripetal and gravitational forces, which operate essentially as bottom-up forces (although this is almost a metaphysical debating point!). Seen from this perspective, the current controversy about ecosystem-level effects of wolf reintroduction to Yellowstone NP is every bit as scientifically exciting as the recent discovery of the Higgs boson. Determining the strength of the forces produced by the loss or addition of particles or species to these very different systems are key scientific questions for the 21st century. Although each discipline uses very different types of equipment, budgets, and collaborations to undertake experiments that generate data for subsequent analysis, they each seek the answer to the same questions: “What are the fundamental forces that structure the universe in which we live, how do they operate, and how can we measure them?”

The hunt for the Higgs boson—an infinitely tiny particle whose energy is required to hold the interior particles of atoms in orbit—was an international research collaboration with a budget that exceeded all funding for ecology over the last ten years, perhaps even over the last century! In contrast, funding for work on natural ecological systems is usually cobbled together from a mixture of government and individual research funds; it is rarely clear from year to year when, or if, funds will appear for the next year's salaries. One benefit of working in national parks is that management occasionally allows experimental introductions, or removals, of species that permit investigation of the impact of these changes at ecosystem-level scales. However, the results of management experiments are rarely clear-cut and often ambiguous. It is all too easy to be critical about lack of controls and absence of replication (which is nontrivially a function of trivial budgets!), but understanding how food webs in national parks react to the addition and loss of species is as scientifically challenging as searching for tiny particles using very expensive particle accelerators. The central problem is ecological budgets are tiny compared to those for “big science,” so we need to use all sources of information that are available, including management exercises, to interpret findings at the appropriate ecosystem-level scale.

If we agree that physicists and ecologists are both trying to understand the forces that determine the structure of the universe, what are the major scientific differences between their approaches? Ecologists are focusing on understanding these forces at the spatial and temporal scales intermediate to that of physicists—less heroic, perhaps, but the scale that is directly relevant to humans. Less heroic or not, from the perspective of systems with interacting components, ecosystems and their constituent species will always be as complicated as those exhibited by atoms and bosons or galaxies and planets, perhaps more so; food webs have many different types of “particles” (species) that interact, evolve, and behave nonlinearly in a huge variety of time, and spatial, scales. Ultimately, we need to arrive at a realization that the mathematics of food webs and ecosystems is as complicated as that found in any of the problems of atomic and galactic structure studied in physics. Increasingly, we are realizing that the quality of human life on the planet depends on a deep functional understanding of the forces that structure the dynamics of food webs and the ecosystem services they provide to the human economy. We may even need new mathematics to deal with these levels and layers of complexity.

The current controversy about the role that wolves play in modifying the behavior and dynamics of other species in Yellowstone is a classic case study in this broader class of problems: it is about understanding how to measure forces and processes that act between operators at a variety of different spatial and temporal rates within a natural ecosystem that contains a diversity of natural heterogeneities (that initially appear to confound the search for broad patterns). If we re-pose ecology as the science that examines the forces that structure the central part of the universe in which we live, then more funding might be available to address these complexities; we would also simultaneously attract more bright minds willing to grapple with complexity.

From a much broader perspective, we need many more ecosystem-level studies of how species interactions between predators, parasites, and prey change the patterns of spatial heterogeneity in vegetation that ultimately drive levels of biodiversity at higher trophic levels. This is an exercise that requires a new generation of spatial, multispecies, multitrophic models and many more debates such as the current one about the role of wolves in Yellowstone. Resolving these discussions will allow ecologists to present a much stronger case to funding agencies and the general public for ecology to be recognized as the central scientific discipline of the 21st century. Ecology's mathematical problems are as complex as anything in physics, and their solutions are required with increasing urgency, particularly if we want to test these assumptions and predictions against viable natural ecosystems.

## References

[1] Ripple WJ, Beschta RL (2012) Trophic cascades in Yellowstone: The first 15years after wolf reintroduction. Biol Conserv 145: 205–213.

[2] Smith DW, Ferguson G (2012) Decade of the Wolf, revised and updated edition: Returning the Wild to Yellowstone. Guilford (Connecticut): Globe Pequot Press.

[3] Vucetich JA, Smith DW, Stahler DR (2005) Influence of harvest, climate and wolf predation on Yellowstone elk, 1961–2004. Oikos 111: 259–270.

[4] Kauffman MJ, Varley N, Smith DW, Stahler D, MacNulty DR, et al (2007) Landscape heterogeneity shapes predation in a newly restored predator–prey system. Ecol Lett 10: 690–700. 17594424 10.1111/j.1461-0248.2007.01059.x

[5] Fortin D, Beyer HL, Boyce MS, Smith DW, Duchesne T, et al (2005) Wolves influence elk movements: Behavior shapes a trophic cascade in Yellowstone National Park. Ecology 86: 1320–1330.

[6] Smith DW, Peterson RO, Houston DB (2003) Yellowstone after wolves. Bioscience 53: 330–340.

[7] Creel S, Christianson DA, Winnie JA (2011) A survey of the effects of wolf predation risk on pregnancy rates and calf recruitment in elk. Ecol Appl 21: 2847–2853.

[8] Creel S, Christianson D, Liley S, Winnie JA (2007) Predation risk affects reproductive physiology and demography of elk. Science 315: 960–960. 17303746 10.1126/science.1135918

[9] Ripple WJ, Beschta RL (2007) Restoring Yellowstone's aspen with wolves. Biol Conserv 138: 514–519.

[10] Hollenbeck JP, Ripple WJ (2008) Aspen snag dynamics, cavity-nesting birds, and trophic cascades in Yellowstone's northern range. For Ecol Manage 255: 1095–1103.

[11] Kauffman MJ, Brodie JF, Jules ES (2010) Are wolves saving Yellowstone's aspen? A landscape-level test of a behaviorally mediated trophic cascade. Ecology 91: 2742–2755. 20957967 10.1890/09-1949.1

[12] David Mech L (2010) Is science in danger of sanctifying the wolf? Biol Conserv 150: 143–149.

[13] Griffin KA, Hebblewhite M, Robinson HS, Zager P, Barber-Meyer SM, et al (2011) Neonatal mortality of elk driven by climate, predator phenology and predator community composition. J Anim Ecol 80: 1246–1257. 21615401 10.1111/j.1365-2656.2011.01856.x

[14] Smith DW, Mech LD, Meagher M, Clark W, Jaffe R (2008) Wolf-bison interactions in Yellowstone National Park. J Mammal 81: 1128–1135.

[15] Williams E, Miller MW (2000) Chronic wasting disease in cervids. Brain Pathol 10: 608–608.

